# Multidimensional Prognostic Index in Association with Future Mortality and Number of Hospital Days in a Population-Based Sample of Older Adults: Results of the EU Funded MPI_AGE Project

**DOI:** 10.1371/journal.pone.0133789

**Published:** 2015-07-29

**Authors:** Sara B. Angleman, Giola Santoni, Alberto Pilotto, Laura Fratiglioni, Anna-Karin Welmer, on behalf of the MPI_AGE Project Investigators

**Affiliations:** 1 Aging Research Center, Department of Neurobiology, Care Sciences and Society (NVS), Karolinska Institutet and Stockholm University, Stockholm, Sweden; 2 Geriatrics Unit, Azienda ULSS 16 Padova, S. Antonio Hospital, Padova, Italy; 3 Department of OrthoGeriatrics, Rehabilitation and Stabilization, Frailty Area, E.O. Galliera Hospital, Genova, Italy; 4 Stockholm Gerontology Research Center, Department of NVS, Karolinska Institutet, Stockholm, Sweden; 5 Karolinska University Hospital, Stockholm, Sweden; University of Padova, ITALY

## Abstract

**Background:**

The Multidimensional Prognostic Index (MPI) has been found to predict mortality in patients with a variety of clinical conditions. We aimed to assess the association of the MPI with future mortality and number of in-hospital days for the first time in a population-based cohort.

**Methods:**

The study population consisted of 2472 persons, aged 66–99 years, from the Swedish National Study on Aging and Care in Kungsholmen, Sweden, who underwent the baseline visit 2001–4, and were followed up >10 years for in-hospital days and >12 years for mortality. The MPI was a modified version of the original and aggregated seven domains (personal and instrumental activities of daily living, cognitive function, illness severity and comorbidity, number of medications, co-habitation status, and nutritional status). The MPI score was divided into risk groups: low, medium and high. Number of in-hospital days (within 1, 3 and 10 years) and mortality data were derived from official registries. All analyses were age-stratified (sexagenarians, septuagenarians, octogenarians, nonagenarians).

**Results:**

During the follow-up 1331 persons (53.8%) died. Laplace regression models, suggested that median survival in medium risk groups varied by age from 2.2–3.6 years earlier than for those in the corresponding low risk groups (p = 0.002-p<0.001), and median survival in high risk groups varied by age from 3.8–9.0 years earlier than for corresponding low risk groups (p<0.001). For nonagenarians, the median age at death was 3.8 years earlier in the high risk group than for the low risk group (p<0.001). The mean number of in-hospital days increased significantly with higher MPI risk score within 1 and 3 years for people of each age group.

**Conclusion:**

For the first time, the effectiveness of MPI has been verified in a population-based cohort. Higher MPI risk scores associated with more days in hospital and with fewer years of survival, across a broad and stratified age range.

## Introduction

Predicting life expectancy is crucial for clinicians to identify the most appropriate clinical decisions for management, treatment and prevention, as well as for patients to have realistic expectations [1, 2]. Several prognostic indices for mortality have been created and validated in different settings, although few scales have been tested for accuracy across settings [3, 4]. In addition, many scales require information that is not routinely collected, often causing their implementation to be time consuming and impractical to use [3]. Finally, older adults often present with several concurrent health problems [5, 6], and life expectancy is likely to be influenced by a multitude of factors, suggesting that a prognostic index for mortality should be multidimensional in nature [7].

The Multidimensional Prognostic Index (MPI) is based on information on functional, cognitive, and nutritional status, as well as medical and social factors, that are usually available in various settings [8]. The MPI was first developed and validated in two independent cohorts of older hospitalized patients [9, 10]. The index has been further validated for all-cause mortality in hospitalized patients with specific acute and chronic diseases [11–15], and in outpatients with cognitive impairment [16]. It has also been associated with mortality in nursing home residents [17]. The MPI has been found to have high predictive validity across these settings, and it has been shown to have high adherence to clinimetric properties, including accuracy, calibration and feasibility. In addition, a large prospective multicenter study found that the MPI had significantly better prognostic accuracy in hospitalized older patients than three other frailty indices in predicting mortality [18].

It has been suggested that older adults who are frequently admitted to hospital constitute a small group that consume a great deal of inpatient care [19]. The MPI has been found to be predictive of in-hospital length of stay and of hospital admissions in an outpatient setting [16, 20]. However, studies have not yet examined the association of the MPI in relation to future mortality and number of in-hospital days in a population-based sample of older adults. Furthermore, most previous studies on prognostic indices for all-cause mortality in older adults, including the MPI, have evaluated mortality risk over time periods ranging from one month to 5 years [3, 4, 9–13, 15, 16]. However, according to current guidelines, some preventive interventions, are not recommended when median life expectancy is less than seven years [21, 22]. Thus, there is a need to use valid predictive tools that can be used to evaluate long-term, in addition to short-term, survival and hospitalization prognosis on a population level. Further, most studies have focused on risk, but have failed to report more familiar and pragmatic measures for both clinicians and patients such as survival time and differences in survival among different risk groups [2, 3].

The purpose of this study was to assess the association of the MPI with future all-cause mortality and number of in-hospital days, for the first time in a population-based cohort. The data regarding these outcomes spanned up to 11–13 years after the assessment of MPI.

## Materials and Methods

### Participants

We used data from the population-based Swedish National Study on Aging and Care in Kungsholmen (SNAC-K) [23]. The SNAC-K study population consists of persons aged 60 years and older living at home or in an institution in the Kungsholmen district of central Stockholm. The study used stratified sampling by age (eleven different age groups: 60, 66, 72, 78, 81, 84, 87, 90, 93, 96, and 99 or older). Of the 5111 persons who were initially selected to be invited for participation, 521 were not eligible (200 died before start of the study, 262 had no contact information, 32 had moved, 23 did not speak Swedish, and 4 were deaf). Among the remaining 4590 persons, 3363 (73.3%) participated at the baseline examination. Baseline survey of all SNAC-K participants was conducted from 2001 through 2004. In this study we excluded: a) 753 participants younger than 66 or older than 99 years, due to power restrictions. There were very few people at age 60 with medium or high risk MPI score (see next paragraph), and few people were 99+ years old; b) 110 participants missing MPI (based on six domains in the primary analyses, exclusive of Mini Nutritional Assessment-Short Form (MNA-SF)); and c) 28 people missing hospital data. This resulted in a study cohort of 2472 people for the primary analyses. In secondary analyses (examining MPI with seven domains, including MNA-SF in the index), an additional 235 participants were excluded due to missing MNA-SF, leaving an analytical sample of 2238 people. As approximately 10% of the study cohort were missing data on MNA-SF, it was decided to test MPI based on six domains, excluding MNA-SF in the primary analyses, yet to repeat all analyses with MPI based on seven domains, including MNA-SF in secondary analyses.

### Ethics

This study was conducted within the SNAC-K, which has the primary aim to increase our understanding of the aging process, and to identify possible preventive strategies to improve health and care in elderly adults. The study was approved by the Regional Ethical Review Board (Regionala Etikprövningsnämnden) in Stockholm, Sweden (number: KI 01–114). Written informed consent was collected from participants. For persons living in institutions for people with dementia, a proxy respondent (a next of kin or a legally authorized representative) was also asked for consent in addition to the participant. If possible, written consent was collected from the proxies. However often proxy consent was obtained via a phone call; in those cases only verbal consent was obtained. When verbal content was obtained, the time and date of the phone call was registered together with the names of the staff who asked for consent and the person who gave the consent. The participant had however always the right to refrain from participating in any or all parts of the study. When visiting the participant, the staff was always attentive to the person's willingness to answer questions or take part in the tests. If, during examination, the participant expressed anguish or discomfort then the interview ended, regardless of whether the person themselves, or a proxy, had given consent. If a person who did not live in an institution and had consented to participate, scored ≤22 on the Mini Mental State Examination (MMSE), a next of kin or a legally authorized representative was contacted to hear that he or she did not oppose the participation. All parts of the study, including the consent procedure, was approved by the Regional Ethical Review Board.

### Data collection

At baseline, data on date of birth, gender, functional, cognitive, and nutritional status, chronic diseases, medication use, and co-habitation status (living with someone, living alone, or being institutionalized) were collected at our research center via interviews and clinical examinations by trained staff. For those who agreed to participate but were unable or unwilling to come to the research center, examinations were conducted at home or institution (n = 706).

Information about the *vital status* of the participants up until end of June 2014 was obtained from the Swedish Death Registry. Information on the *total number of hospital days in inpatient care* between the baseline examination and up until the end of January 2012 was retrieved from the National Inpatient Registry.

*Functional status* comprised two domains of the MPI and was evaluated by number of personal or instrumental Activities of Daily Living (P-ADL and I-ADL) that the participants managed independently. P-ADL included six basic daily activities (dressing, hygiene, bathing, feeding, continence, and transferring), and I-ADL included eight activities (managing finances, taking medications, using telephone, shopping, using transportation, preparing meals, cleaning, and washing). For IADL, participants were coded as independent if they stated that they could do an activity, even if they never did, e.g. cleaning.

*Global cognitive functioning* was assessed with the MMSE, instead of the short portable mental status questionnaire (SPMSQ), as the latter instrument was not available in SNAC-K. The MMSE is a commonly used screening test for dementia, and measures basic cognitive domains such as orientation, memory, attention, language, visuospatial, and executive functioning (scores ranging from 0–30, and 30 representing the best performance) [24]. A significant correlation between the MMSE and the SPMSQ has been reported [25].

*Nutritional status* was defined based on the Mini Nutritional Assessment-Short Form (MNA-SF) [26]. The MNA-SF consists of six items from the full MNA, including declining food intake, weight loss, mobility problems, psychological stress or acute disease, neuropsychological problems such as dementia and depression, and body mass index (BMI). The MNA-SF features the same three categories as the full 18 item MNA (malnourished, at risk, and well nourished) and has shown 90.8% agreement with the full MNA in the community-setting [27]. In this study, we used the MNA-SF, instead of the complete MNA, as in the original MPI [10]. A previous study on elderly hospitalized patients however found a MPI that included the MNA-SF to be equally predictive of mortality as a MPI that included the full MNA [9].

*Medical diagnoses* were made by the examining physicians based on the clinical examination, medical history and laboratory data. Illness severity and comorbidity was assessed using the Cumulative Illness Rating Scale (CIRS) [28], a standardized summary score which includes the physician’s ratings of the degree of pathology in each of the following 13 major organ systems: cardiac, hypertension, vascular, respiratory, eye-ear-nose-throat, lower gastrointestinal, hepatic, renal, other genitourinary, musculoskeletal, neurological, endocrine-metabolic, and psychiatric behavioral disorders. The severity of impairment in each organ system is rated from 1 = none to 5 = extremely severe (impairment is life threatening). The CIRS score (possible range 0–13) was transformed into the Comorbidity Index (CIRS-CI) to reflect the number of organ groups with at least moderate levels of pathology (grades 3–5) [28].

*Medication use* was evaluated by number of regularly consumed, prescribed drugs (nutritional supplements were not included) at the baseline examination. Information on medication use was collected by the physician during the clinical examination. For cognitively impaired persons, a proxy or caregiver was asked instead. Before the interview, participants were instructed to bring a list of currently used medications. If the person was living in a nursing home, information on medication use was collected directly from medical records. The Anatomical Therapeutic Chemical (ATC) classification system was used to classify medications [29].

### Calculation of the MPI

We calculated the MPI as established in previous studies, with some modifications based on availability of data. We used seven of the original MPI domains: P-ADL, I-ADL, cognitive function (MMSE), illness severity and comorbidity (CIRS), the number of medications, and co-habitation status. As stated previously, approximately 10% of the study cohort was missing nutritional status via MNA-SF, so we calculated this index both excluding (primary analyses) and including (supplementary secondary analyses) this item. We also lacked pressure sore risk via the Exton-Smith scale [10]. For each of the seven domains, a three-level score was assigned with score 0 indicating low risk of mortality and/or longer hospitalization, score 0.5 indicating a middle level of risk, and score 1 indicating high risk, as established previously in previous studies [9, 10]. Each domain was categorized as follows: *number of independent P-ADL*: 5–6 = score 0, 3–4 = score 0.5, and 0–2 = score 1; *number of independent I-ADL*: 6–8 = score 0, 4–5 = score 0.5, and 0–3 = score 1; *MMSE score [30]*: 28–30 = score 0, 25–27 = score 0.5, and 0–24 = score 1; *CIRS score*: 0 = score 0, 1–2 = score 0.5, and ≥3 = score 1; *number of medications*: 0–3 = score 0, 4–6 = score 0.5, and ≥7 = score 1; *co-habitation status*: living with someone = score 0, being institutionalized = score 0.5, and living alone = score 1; and *MNA-SF score*: ≥12 (well nourished) = score 0, 8–11 (at risk) = score 0.5, and 0–7 (malnourished) = score 1 [9, 10]. The sum of the calculated scores was then divided by seven (when MNA-SF was included) or by 6 (when MNA-SF was excluded) to obtain a MPI risk score ranging between 0 and 1. As our aim was to verify the effectiveness of the previously established index in this population-based cohort, we used the previously defined cut points: MPI scores 0–0.33 were considered low risk, MPI scores 0.34–0.66 were considered medium risk, and MPI scores 0.67–1.0 were considered high risk [9, 10].

### Data analysis

Mean number of hospital days (within 1, 3 and 10 years from baseline visit) and 95% confidence intervals were calculated, by age group and MPI level, and quantitative tests for trend were performed across levels of MPI within each age group. Median time to death for medium and high MPI risk groups and 95% confidence intervals were calculated, in comparison to the MPI low risk group using Laplace regression [31], in three separate sets of models: unadjusted, adjusted only for age, and adjusted for age and gender. All analyses were stratified by four age groups: sexagenarians (age cohort 66), septuagenarians (age cohorts 72 and 78), octogenarians (age cohorts 81, 84 and 87) and nonagenarians (age cohorts 90, 93, 96, 99). This age stratification was necessary because as expected, both MPI and number of hospital days and time to death were highly correlated with age. However, the Laplace regression mortality analyses excluded sexagenarians as too few of this age group had died by the censoring date for mortality. Statistical analyses were performed with STATA/SE 13.1 software (Texas, USA). Statistical significance was based on p-values <0.05.

## Results

During the follow-up 1331 persons (53.8%) died (maximum follow-up for mortality 12.8 years). Characteristics of the study cohort are presented in Table 1, stratified by age group. As expected, having a medium or high risk level of MPI increased dramatically with age. Nearly all sexagenarians had a low risk level of MPI (93.9%), which declined to 83.1% of septuagenarians, 59.0% of octogenarians, and only 28.0% of nonagenarians (Table 1). Having a high risk level of MPI was uncommon in this population-based sample, extremely so in the younger two age groups, and reaching only 5% and 16% respectively amongst the octogenarians and nonagenarians. The mean number of days spent in hospital during the follow-up period was lowest amongst the sexagenarians, but did not substantially differ amongst the oldest three age groups (Table 1).

**Table 1:** Characteristics of the Study Cohort.

	66		72–78		81–87		90–99	
**N (Total = 2472)**	554		904		600		414	
**Women (%)**	57.9		63.5		70.0		82.4	
**MPI** *								
**Low risk % (N)**	93.9	(520)	83.1	(751)	59.0	(354)	28.0	(116)
**Medium Risk % (N)**	6.0	(33)	15.9	(144)	36.0	(216)	56.0	(232)
**High risk % (N)**	0.2	(1)	1.0	(9)	5.0	(30)	16.0	(66)
**Number of in-hospital days** ^**ǂ**^								
**Mean (95% CI)**	18.9 (15.1–22.6)		41.4 (36.9–46.0)		55.2 (49.9–60.5)		46.8 (42.6–50.9)	
**Deaths** ^**ǂ**^ **(%)**	18.1		41.9		75.7		96.1	

*Multidimensional Prognostic Index (MPI) aggregated six domains (personal and instrumental activities of daily living, cognitive function, illness severity and comorbidity, the number of medications, co-habitation status).

ǂA total follow-up time for mortality of a maximum of 12.8 years and for number of in-hospital days of a maximum of 10.8 years.

Mean number of days spent in hospital within one year of the baseline visit were significantly associated with MPI level of risk, for each age group (Table 2). Similarly and consistently, significant findings were found for number of days spent in hospital within three years of the baseline visit. Findings were likewise similar even within 10 years from the baseline visit, although the association was attenuated in significance and linearity, with the highest number of hospital days being found in the moderate MPI risk category.

**Table 2:** Mean Number of In-Hospital Days within 1, 3 and 10 Years Since Baseline, by Multidimensional Prognostic Index (MPI) Status and Age.

	66	72–78	81–87	90–99
**Mean number of in-hospital days (95% CI) within 1 year of baseline visit**				
**MPI**				
**Low Risk**	2.9 (1.8–3.9)	4.3 (3.3–5.3)	7.8 (3.5–12.1)	11.0 (7.4–14.5)
**Medium Risk**	9.0 (3.3–14.6)	13.3 (9.0–17.6)	18.9 (14.7–23.1)	24.5 (20.7–28.3)
**High Risk**	—-	30.1 (6.3–53.9)	28.2 (16.3–40.0)	30.7 (22.4–39.0)
**p**	<0.001	<0.001	<0.001	<0.001
**Mean number of in-hospital days (95% CI) within 3 years of baseline visit**				
**MPI**				
**Low Risk**	6.4 (4.5–8.3)	9.6 (8.0–11.2)	16.3 (11.3–21.4)	24.3 (19.3–29.3)
**Medium Risk**	14.6 (6.5–22.7)	27.4 (21.2–33.6)	34.0 (27.8–40.1)	37.1 (32.2–42.0)
**High Risk**	—-	33.9 (6.8–61.0)	41.3 (21.0–61.6)	36.9 (26.4–47.3)
**p**	<0.001	<0.001	<0.001	0.006
**Mean number of in-hospital days (95% CI) within 10 years of baseline visit**				
**MPI**				
**Low Risk**	17.4 (13.5–21.2)	37.9 (32.8–42.9)	48.7 (41.9–55.4)	46.8 (39.8–53.8)
**Medium Risk**	35.5 (19.6–51.4)	58.5 (48.0–69.0)	66.5 (57.2–75.8)	49.0 (43.3–54.6)
**High Risk**	—-	34.6 (6.8–62.3)	45.7 (25.1–66.2)	37.9 (27.0–48.9)
**p**	<0.001	0.005	0.006	0.176

Multidimensional Prognostic Index (MPI) aggregated six domains (personal and instrumental activities of daily living, cognitive function, illness severity and comorbidity, the number of medications, co-habitation status). Age group 66 with high risk MPI omitted, due to only one participant in this category.

The median survival of people in medium risk MPI groups varied across age groups from 2.2–3.6 years earlier than for those in the low risk MPI group (p = 0.002-p<0.001), and the median survival of people in high risk MPI groups varied by age from 3.8–9.0 years earlier than for those in the low risk MPI group (p<0.001) (Table 3). Even among nonagenarians, the median age at death was 3.8 years earlier for those in the high risk MPI group than for those in the low risk MPI group (p<0.001). Controlling for age, or for age and gender, did not substantially change the findings.

**Table 3:** Median Time to Death in Years, by Multidimensional Prognostic Index (MPI) Status and Age.

	72–78			81–87			90–99		
**Unadjusted**									
**MPI**	**Years**	**95% CI**	**p**	**Years**	**95% CI**	**p**	**Years**	**95% CI**	**p**
**Low Risk**	Ref			Ref			Ref		
**Medium Risk**	-2.6	-4.2–-0.9	0.002	-3.6	-4.5–-2.8	<0.001	-2.2	-3.7–-0.7	0.005
**High Risk**	-9.0	-10.0–-8.1	<0.001	-7.2	-8.8–-5.6	<0.001	-3.8	-5.3–-2.3	<0.001
**Adjusted for age**									
**MPI**	**Years**	**95% CI**	**p**	**Years**	**95% CI**	**p**	**Years**	**95% CI**	**p**
**Low Risk**	Ref			Ref			Ref		
**Medium Risk**	-2.7	-4.3–-1.1	0.001	-3.3	-4.1–-2.6	<0.001	-1.6	-2.8–-0.4	0.01
**High Risk**	-8.8	-10.3–-7.4	<0.001	-6.6	-7.5–-5.7	<0.001	-3.1	-4.3–-1.9	<0.001
**Adjusted for age and gender**									
**MPI**	**Years**	**95% CI**	**p**	**Years**	**95% CI**	**p**	**Years**	**95% CI**	**p**
**Low Risk**	Ref			Ref			Ref		
**Medium Risk**	-2.5	-4.4–-0.6	0.009	-3.6	-4.3–-2.8	<0.001	-2.2	-3.1–-1.3	<0.001
**High Risk**	-8.9	-10.4–-7.5	<0.001	-6.8	-7.6–-5.9	<0.001	-3.8	-4.7–-2.8	<0.001

Multidimensional Prognostic Index (MPI) aggregated six domains (personal and instrumental activities of daily living, cognitive function, illness severity and comorbidity, the number of medications, co-habitation status). Age 66 excluded because too few had died to estimate median time to death. Mortality data until 2014-06-26. Analysis used Laplace regression.

Sensitivity analyses repeated all of the main analyses using a differently calculated MPI variable, which included nutritional status (using MNA-SF) (Tables A, B and C in S1 File). As MNA-SF was missing in approximately 10% of the study population, primary analyses were conducted using MPI without MNA, to maximize power and size of the analytical sample. Generally, findings were substantially unchanged. However, there were no septuagenarians with a high level of MPI risk. Sensitivity analyses were also conducted stratifying by gender, and stratifying by dementia status (Table D in S1 File), for the oldest two age groups (this was not possible in the younger age groups, due to insufficient power for further stratification beyond age). The results were substantially unchanged from the primary analyses presented in Table 3, except for shorter median time to death for persons with dementia in the high risk MPI group, and loss of significance in the medium risk MPI group, which is at least partially due to insufficient power, as most persons with dementia would have had high risk MPI.

## Discussion

In this population-based cohort, a modified version of the MPI has been found to associate both with future number of days in hospital and with mortality, across a broad age span from 66–99 years. Although having a high level of MPI risk was relatively uncommon in this population-based cohort, it was still significantly associated with spending a greater number of days in hospital and a shorter time to death. Having a moderate level of MPI risk was not uncommon, particularly amongst octogenarians and nonagenarians (more than a third and a half of each age group respectively). The moderate level of MPI risk was also significantly associated with spending a greater number of days in hospital and a shorter time to death. Our results suggest a potential use for applying this prognostic index in the general population, in order to identify an at-risk segment of the population that may benefit from intervention or for public health planning of additional health care resources. The relatively large proportion of sexagenarians and septuagenarians in the low risk category (94% of sexagenarians and 83% of septuagenarians), however indicates that the MPI may be most useful to apply amongst octogenarians and nonagenarians. Furthermore, our results indicate that MPI is most strongly predictive of future number of in-hospital days within shorter lengths of follow-up (≤3 years) than within longer lengths of follow-up (10 years), possibly due to the higher mortality risk amongst persons in the medium and high MPI risk categories.

Previous studies have also found prognostic associations of MPI with hospitalizations and with mortality, however they were not based on population-based cohorts, and they did not look at long-term number of days in hospital or mortality [3, 9–13, 15, 16, 18, 20]. One recent study evaluated an instrument related to the MPI in community-dwelling older adults, called the MPI-SVaMA (a Multidimensional Prognostic Index for Mortality based on a Standardized Multidimensional Assessment Schedule) [32]. This instrument varied substantially from the standard MPI instrument, and it was evaluated only in relation to short-term mortality (1 month and 1 year). However, it was also found to be an effective prognostic tool for short-term mortality, which substantiates the flexibility and strength of even modified forms of the MPI as a tool that can be used in community-based settings, as well in hospital or institutional settings. However, this study selected participants to the cohort based on the requirement that they required public health care intervention or support, so they were likely not as healthy as the population-based cohort used in the present study, which was selected based on geography (and included people both living at home and in institutions within the geographically-defined area) not on care needs. An advantage of the MPI is that it is based on commonly assessed measures of morbidity, disability, cognitive, and nutritional status, and social factors. However, several other prognostic indices for all-cause mortality have been proposed, with substantive differences in their ability to predict mortality [3, 4]. Future studies should compare the predictive ability of the MPI with other mortality indices in population-based studies of older adults.

There are some limitations to this analysis. The MPI instrument investigated in the present study is a modified version of the original. First, the Exton-Smith Scale was excluded, as this data was not available in SNAC-K. In the development study of the original MPI on hospitalized older patients, the Exton-Smith Scale was shown to be the strongest individual predictor of mortality. However, the prognostic value of the MPI was higher compared to those of the individual components [10]. In addition, as the Exton-Smith Scale is an instrument to predict risk of bed sores, this is not likely a risk that is impacting the vast majority of this population-based cohort. Second, we used MMSE instead of the SPMSQ, as the latter instrument was not available in SNAC-K. However, MMSE is a well-validated measure of cognitive status, and a significant correlation with the SPMSQ has been reported [25]. Finally, our primary analysis did not include MNA in the MPI instrument, as MNA was missing in approximately 10% of the SNAC-K study cohort, and we wanted to maximize power. However, we did conduct secondary analyses repeating all the primary analyses performed, which included MNA-SF in the MPI, and findings were substantially unchanged. Also, in the secondary analyses, we used MNA-SF instead of the full MNA, as the latter was not available in SNAC-K. A previous study on elderly hospitalized patients found a MPI that included the MNA-SF to be equally predictive of mortality as a MPI that included the full MNA [7]. Despite these modifications, MPI was still found to be associated with both short and long-term outcomes of mortality and number of in-hospital days. The ability of this index to associate with future number of in-hospital days and death, even when using a modified version of the instrument, is a strength of the index, as it suggests it is sufficiently generalizable and flexible for implementation in various settings, which will possibly lack easy access to one or more components of the MPI, or will have variation in some of the items included in the instrument. Finally, for IADL the same cut points are used for men and women in the MPI [9, 10], which may create a potential bias. However, participants were coded as independent if they stated that they could do an activity, even if they never did. In addition controlling for gender or stratifying by gender did not substantially change the results of the analyses.

For the first time, the effectiveness of this modified version of the MPI has been verified in a population-based cohort, and across a broad and stratified age range. Higher MPI risk scores associated with more days in hospital within one and three years of the baseline examination and with fewer years of survival. Furthermore, our results indicate that the MPI may be particularly useful among octogenarians and nonagenarians.

## Supporting Information

S1 File MPI_PLOS ONE_Angleman_Supporting Information_Tables. Table A. Characteristics of the Study Cohort, using MPI including MNA-SF. Table B. Mean Number of In-Hospital Days within 1, 3and 10 Years Since Baseline, by Multidimensional Prognostic Index (MPI) Status and Age, using MPI including MNA-SF. Table C. Median Time to Death in Years, by MPI Status and Age, using MPI including MNA-SF. Table D. Median Time to Death in Years, by Multidimensional Prognostic Index (MPI) Status and Age, stratified by gender or by dementia status.
(DOCX)
