# Supplementary material for: Multidimensional Prognostic Index in Association with Future Mortality and Number of Hospital Days in a Population-Based Sample of Older Adults: Results of the EU Funded MPI_AGE Project
Source: PLoS One. 2015 Jul 29;10(7):e0133789. doi: 10.1371/journal.pone.0133789 (PMC4519042; doi:10.1371/journal.pone.0133789)
Supplement: S1 File — Table A. Characteristics of the Study Cohort, using MPI including MNA-SF. Table B. Mean Number of In-Hospital Days within 1, 3and 10 Years Since Baseline, by Multidimensional Prognostic Index (MPI) Status and Age, using MPI including MNA-SF. Table C. Median Time to Death in Years, by MPI Status and Age, using MPI including MNA-SF. Table D. Median Time to Death in Years, by Multidimensional Prognostic Index (MPI) Status and Age, stratified by gender or by dementia status. (DOCX) [file pone.0133789.s001.docx]

**Table A. Characteristics of the Study Cohort, using MPI including MNA-SF**

|  | **66** | | **72-78** | | **81-87** | | **90-99** | |
| --- | --- | --- | --- | --- | --- | --- | --- | --- |
| **N (Total=2238)** | 537 | | 859 | | 536 | | 306 | |
| **Women %** | 58.8 | | 63.1 | | 70.1 | | 79.7 | |
| **MPI*** |  | |  | |  | |  | |
| **Low risk % (N)** | 92.6 | (497) | 80.8 | (694) | 56.5 | (303) | 28.1 | (86) |
| **Medium risk % (N)** | 7.3 | (39) | 19.2 | (165) | 40.3 | (216) | 66.0 | (202) |
| **High risk % (N)** | 0.2 | (1) | 0 | (0) | 3.2 | (17) | 5.9 | (18) |
| **Number of in-hospital days**^ǂ^ |  | |  | |  | |  | |
| **Mean (95% CI)** | 18.5 (14.7-22.4) | | 41.1 (36.4-45.8) | | 55.0 (49.8-60.2) | | 52.4 (47.7-57.1) | |
| **Number of deaths**^ǂ^ **(%)** | 17.5 | | 41.0 | | 73.9 | | 94.8 | |

*Multidimensional Prognostic Index (MPI) aggregated seven domains (personal and instrumental activities of daily living, cognitive function, illness severity and comorbidity, the number of medications, co-habitation status, nutritional status). ǂA total follow-up time for mortality of a maximum of 12.8 years and for hospitalizations of a maximum of 10.8 years.

**Table B. Mean Number of In-Hospital Days within 1, 3and 10 Years Since Baseline, by Multidimensional Prognostic Index (MPI) Status and Age, using MPI including MNA-SF**

|  | **66** | **72-78** | **81-87** | **90-99** |
| --- | --- | --- | --- | --- |
| **Mean number of in-hospital days (95% CI) within 1 year of baseline visit** | | | | |
| **MPI*** | | | | |
| **Low Risk** | 2.7 (1.6-3.8) | 4.1 (3.1-5.1) | 5.0 (3.7-6.3) | 10.0 (6.0-13.9) |
| **Medium Risk** | 6.8 (2.5-11.1) | 11.6 (7.7-15.5) | 16.3 (12.5-20.2) | 25.6 (21.5-29.8) |
| **High Risk** | --- | --- | 43.3 (22.2-64.4) | 39.0 (17.0-61.0) |
| **p** | <0.001 | <0.001 | <0.001 | <0.001 |
| **Mean number of in-hospital days (95% CI) within 3 years of baseline visit** | | | | |
| **MPI*** | | | | |
| **Low Risk** | 6.1 (4.2-8.1) | 9.2 (7.5-10.8) | 13.6 (10.4-16.8) | 24.0 (18.4-29.6) |
| **Medium Risk** | 13.2 (5.8-20.7) | 23.4 (17.8-28.9) | 30.4 (24.8-36.1) | 40.2 (34.8-45.6) |
| **High Risk** | --- | --- | 65.6 (31.8-99.4) | 44.0 (18.3-69.7) |
| **p** | 0.001 | <0.001 | <0.001 | 0.002 |
| **Mean number of in-hospital days (95% CI) within 10 years of baseline visit** | | | | |
| **MPI*** | | | | |
| **Low Risk** | 17.0 (13.1-21.0) | 37.6 (32.2-43.0) | 45.6 (39.6-51.6) | 48.5 (41.1-55.9) |
| **Medium Risk** | 31.7 (19.0-44.3) | 54.6 (45.7-63.5) | 66.2 (57.0-75.4) | 54.4 (48.4-60.4) |
| **High Risk** | --- | --- | 73.3 (40.6-106.0) | 44.9 (18.4-71.5) |
| **p** | 0.001 | 0.005 | <0.001 | 0.415 |

*Multidimensional Prognostic Index (MPI) aggregated seven domains (personal and instrumental activities of daily living, cognitive function, illness severity and comorbidity, the number of medications, co-habitation status, nutritional status). Age group 66 with high risk MPI omitted, due to only one participant in this category. Age group 72-78 with high risk MPI omitted, because no participants in this category.

**Table C. Median Time to Death in Years, by MPI Status and Age, using MPI including MNA-SF**

|  | **72-78** | | | **81-87** | | | **90-99** | | |
| --- | --- | --- | --- | --- | --- | --- | --- | --- | --- |
| **Unadjusted** |  | |  |  | |  |  | |  |
| **MPI*** | **Years** | **95% CI** | **p** | **Years** | **95% CI** | **p** | **Years** | **95% CI** | **p** |
| **Low Risk** | Ref |  |  | Ref |  |  | Ref |  |  |
| **Medium Risk** | -1.9 | -3.2 - -0.7 | 0.002 | -3.6 | -4.7 - -2.4 | <0.001 | -1.7 | -3.3 - -0.2 | 0.031 |
| **High Risk** | --- | --- | --- | -6.8 | -8.6 - -5.1 | <0.001 | -3.8 | -5.5 - -2.1 | <0.001 |
| **Adjusted for age** | | | | | | | | | |
| **MPI*** | **Years** | **95% CI** | **p** | **Years** | **95% CI** | **p** | **Years** | **95% CI** | **p** |
| **Low Risk** | Ref |  |  | Ref |  |  | Ref |  |  |
| **Medium Risk** | -1.6 | -2.9 - -0.4 | 0.011 | -3.1 | -4.1 - -2.1 | <0.001 | -1.0 | -2.4 – 0.3 | 0.136 |
| **High Risk** | --- | --- | --- | -6.3 | -7.4 - -5.1 | <0.001 | -2.9 | -4.3 - -1.4 | <0.001 |
| **Adjusted for age and gender** | | | | | | | | | |
| **MPI*** | **Years** | **95% CI** | **p** | **Years** | **95% CI** | **p** | **Years** | **95% CI** | **p** |
| **Low Risk** | Ref |  |  | Ref |  |  | Ref |  |  |
| **Medium Risk** | -1.8 | -3.0 - -0.7 | 0.001 | -3.2 | -4.1 - -2.2 | <0.001 | -1.9 | -2.8 - -1.0 | <0.001 |
| **High Risk** | --- | --- | --- | -6.6 | -7.5 - -5.7 | <0.001 | -3.3 | -4.9 - -1.6 | <0.001 |

*Multidimensional Prognostic Index (MPI) aggregated seven domains (personal and instrumental activities of daily living, cognitive function, illness severity and comorbidity, the number of medications, co-habitation status, nutritional status). Age 66 excluded because too few had died to estimate median time to death. Mortality data until 2014-06-26. Analysis used Laplace regression.

**Table D. Median Time to Death in Years, by Multidimensional Prognostic Index (MPI) Status and Age, stratified by gender or by dementia status**

|  |  | | | **81-87** | | | **90-99** | | |
| --- | --- | --- | --- | --- | --- | --- | --- | --- | --- |
| **Men only** |  | |  | **N=180** | |  | **N=73** | |  |
| **MPI** |  |  |  | **Years** | **95% CI** | **P** | **Years** | **95% CI** | **p** |
| **Low Risk** |  |  |  | Ref |  |  | Ref |  |  |
| **Medium Risk** |  |  |  | -3.5 | -4.9 - -2.2 | <0.001 | -1.6 | -2.9 - -0.3 | 0.013 |
| **High Risk** |  |  |  | -7.4 | -8.6 - -6.1 | <0.001 | -3.2 | -4.6 - -1.7 | <0.001 |
| **Women only N=420 N=341** | | | | | | | | | |
| **MPI** |  |  |  | **Years** | **95% CI** | **P** | **Years** | **95% CI** | **P** |
| **Low Risk** |  |  |  | Ref |  |  | Ref |  |  |
| **Medium Risk** |  |  |  | -4.0 | -4.9 - -3.0 | <0.001 | -2.5 | -3.6 - -1.5 | <0.001 |
| **High Risk** |  |  |  | -7.8 | -9.3 - -6.3 | <0.001 | -3.1 | -5.4 - -3.3 | <0.001 |
| **Dementia only N=75 N=142** | | | | | | | | | |
| **MPI** |  |  |  | **Years** | **95% CI** | **P** | **Years** | **95% CI** | **P** |
| **Low Risk** |  |  |  | Ref |  |  | Ref |  |  |
| **Medium Risk** |  |  |  | -1.1 | -2.7 – 0.5 | 0.181 | -1.6 | -3.6 - 0.3 | 0.093 |
| **High Risk** |  |  |  | -3.1 | -4.4 - -1.7 | <0.001 | -2.6 | -4.4 - -0.7 | 0.006 |
| **No Dementia only N=525 N=272** | | | | | | | | | |
| **MPI** |  |  |  | **Years** | **95% CI** | **P** | **Years** | **95% CI** | **P** |
| **Low Risk** |  |  |  | Ref |  |  | Ref |  |  |
| **Medium Risk** |  |  |  | -3.4 | -4.4 - -2.4 | <0.001 | -1.5 | -2.3 - -0.2 | 0.021 |
| **High Risk** |  |  |  | -6.6 | -8.2 - -5.0 | <0.001 | -2.1 | -4.1 - -0.004 | 0.050 |

Multidimensional Prognostic Index (MPI) aggregated six domains (personal and instrumental activities of daily living, cognitive function, illness severity and comorbidity, the number of medications, co-habitation status). Age 66 excluded because too few had died to estimate median time to death. Age 72,78 excluded because not enough power for further stratification by gender or dementia status. Mortality data until 2014-06-26. Analysis used Laplace regression.
